# Irinotecan+5-fluorouracil with concomitant pre-operative radiotherapy in locally advanced non-resectable rectal cancer: a phase I/II study

**DOI:** 10.1038/sj.bjc.6604292

**Published:** 2008-03-18

**Authors:** S M Iles, S W Gollins, S Susnerwala, B Haylock, S Myint, A Biswas, R Swindell, E Levine

**Affiliations:** 1 Department of Clinical Oncology, The Christie Hospital NHS Trust, Manchester M20 4BX, UK; 2 Department of Clinical Oncology, North Wales Cancer Treatment Centre, Rhyl LL18 5UJ, UK; 3 Department of Clinical Oncology, Royal Preston Hospital, Preston PR2 9HT, UK; 4 Department of Clinical Oncology, Clatterbridge Centre for Oncology, Liverpool CH63 4JY, UK; 5 Department of Medical Statistics, The Christie Hospital NHS Trust, Manchester M20 4BX, UK

**Keywords:** chemotherapy, combined modality, irinotecan, locally advanced, radiation, rectal cancer

## Abstract

In the UK, 10% of patients diagnosed with rectal cancer have inoperable disease at presentation. This study ascertained whether the resectability rate of inoperable locally advanced rectal cancer was improved by administration of intravenous irinotecan, 5-fluorouracil (5-FU) and pelvic radiotherapy. During phase I of the trial (*n*=12), the dose of irinotecan was escalated in three-patient cohorts from 50 mg m^−2^ to 60 mg m^−2^ to 70 mg m^−2^ to identify the maximum tolerated dose (60 mg m^−2^). In phase II, 31 patients with non-resectable disease received 45 Gy radiotherapy and 5-FU infusions (200 mg m^−2^ per day) for 5 weeks. Irinotecan (60 mg m^−2^) was given on days 1, 8, 15 and 22. After treatment, patients were operated on if possible. Thirty patients completed the protocol, 28 underwent surgery. Before surgery, MRI restaging of 24 patients showed that 19 (79%) had a reduction in tumour stage after treatment (seven complete clinical response and 12 partial). Of 27 patients followed up after surgery, 22 (81%) had clear circumferential resection margins. Disease-free and overall survival estimates at 3 years were 65 and 90%, respectively. The regimen was well tolerated. Irinotecan, 5-FU and radiotherapy results in tumour downgrading, allowing resection of previously inoperable tumour with acceptable toxicity.

More than 13 000 new cases of rectal cancer are diagnosed every year in the UK (Office for National Statistics, 2003; Northern Ireland Cancer Registry, 2007; Welsh Cancer Intelligence and Surveillance Unit, 2007). At least 10% of these cancers are inoperable because of involvement of, or penetration through, the mesorectal fascia and implication of other organs (Devita *et al*, 2001). For patients with inoperable disease, prognosis is poor. Radiotherapy and, more recently, combined chemo-radiotherapy (CRT) regimens have been shown to be able to reduce the stage and size of advanced tumours, making them amenable to resection. The ideal combined-modality pre-operative regimen is, however, yet to be determined.

To date, phase I/II trials have assessed the effect of radiation in combination with 5-fluorouracil (5-FU) or another fluoropyrimidine with or without leucovorin (to enhance the actions of 5-FU) (Videtic *et al*, 1998; Rodel *et al*, 2000). Post-operative chemotherapy is also sometimes included in the CRT regimen (Minsky *et al*, 1993). Overall, results from these studies have been favourable, resulting in the widespread adoption of CRT regimens, mostly based on 5-FU, in the neoadjuvant treatment of inoperable rectal cancer.

Recently, several novel therapies have emerged with activity in rectal cancer. These include raltitrexed, irinotecan, oxaliplatin and oral fluoropyrimidines, including uracil-tegafur and capecitabine (de la Torre *et al*, 1999; Kalofonos *et al*, 2003; Fernandez-Martos *et al*, 2004; Gambacorta *et al*, 2004a, 2004b; Hofheinz *et al*, 2005; Klautke *et al*, 2005, 2006). Despite the advent of oral fluoropyrimidines, intravenous infusion remains popular in Europe and the United States, because nausea, vomiting and diarrhoea can affect compliance and absorption of oral drugs (Minsky *et al*, 1993; Rodel *et al*, 2000; Mohiuddin *et al*, 2006). For metastatic colorectal cancer, the addition of irinotecan to treatment with 5-FU and leucovorin significantly improves progression-free and overall survival (Douillard *et al*, 2000; Saltz *et al*, 2000). This finding has resulted in an interest in developing its use as part of a combined protocol for the treatment of locally advanced disease.

Several phase I and phase II trials have investigated the use of irinotecan as a radiation sensitizer in CRT regimens for locally advanced rectal cancer; however, results are mostly published as meeting abstracts, with short-term follow up only. Irinotecan has been administered as monotherapy combined with radiation, but most of the studies have assessed the CRT regimen of irinotecan plus 5-FU and radiotherapy (Minsky *et al*, 1999; Klautke *et al*, 2001, 2005, 2006; Kalofonos *et al*, 2003; Mehta *et al*, 2003; Navarro *et al*, 2003, 2007; Mitchell *et al*, 2004; Sebag-Montefiore *et al*, 2005).

Overall, the results of these preliminary studies suggest promising activity of irinotecan-containing regimens. Six small studies are published as full papers (Kalofonos *et al*, 2003; Mehta *et al*, 2003; Klautke *et al*, 2005; Mohiuddin *et al*, 2006; Glynne-Jones *et al*, 2007; Willeke *et al*, 2007). To conclude, the combined RCT regimen irinotecan plus 5-FU with radiation therapy appears to be a tolerable adjuvant therapy (Kalofonos *et al*, 2003) that results in a good tumour response when given pre-operatively to patients with rectal cancer in whom resection is either possible or uncertain (Mehta *et al*, 2003; Klautke *et al*, 2005).

This current study was undertaken to rigorously assess and provide long-term follow-up data for the use of irinotecan in combination with 5-FU and radiotherapy for inoperable, locally advanced (T3/T4) rectal tumours. The aims of this study are two fold: (a) to establish a safe dose of intravenous (iv) irinotecan for administration in combination with a standard course of 5-FU and concomitant pelvic radiotherapy to patients with locally advanced non-resectable rectal cancer and (b) to assess the effectiveness of the regimen, in terms of post-treatment resectability of tumours, and disease-free and overall survival.

## MATERIALS AND METHODS

### Participants

Patients with unresectable rectal cancer were recruited between September 2001 and December 2003 from four centres in the UK: the Christie Hospital NHS Trust, Manchester; Clatterbridge Hospital, Liverpool; North Wales Cancer Treatment Centre, Rhyl; and the Royal Preston Hospital, Preston.

The tumour stage of all patients was determined clinically after examination under general anaesthetic and following computed tomography (CT) of the chest, abdomen and pelvis, or magnetic resonance imaging (MRI) of the abdomen or pelvis. In cases of discrepancy, the highest tumour stage was used. All recruited patients were diagnosed as having non-resectable disease, defined as involving the edge of the mesorectal fascia or adherence of the tumour to an adjacent structure or organ, preventing an attempt at resection with a negative circumferential resection margin (CRM). The patients had a WHO performance score of less than two and adequate hepatic and renal function (bilirubin, creatinine, aspartate aminotransferase or alanine aminotransferase <2.5 × upper limit of normal) and bone marrow reserve (absolute neutrophil count >2.0 × 10^9^; platelets >100 × 10^9^; haemoglobin >10.0 g l^−1^).

Patients were excluded if they had received previous radiotherapy or chemotherapy, had been diagnosed with metastatic disease or had a past or current malignancy at other sites (with the exception of adequately treated *in-situ* carcinoma of the cervix uteri and non-melanoma carcinoma of the skin).

All patients provided written informed consent and the study was performed in accordance with the ethical principles outlined in the declaration of Helsinki. Ethical approval for this study was obtained from the ethics committees of all participating centres.

### Procedure

Radiotherapy was given by planned target volume to the pelvic area, treating with 25 fractions at 1.8 Gy/fraction via four fields, with all fields treated daily (45 Gy total dose). The planned target volume was defined using simulator or CT planning. The planned target volume was to include: 3 cm superior, inferior and lateral of the extent of the gross tumour volume, but no higher than the sacral promontory; the posterior border of the most posterior aspect of the sacrum; and 2 cm anterior to the tumour or the anterior rectal wall, whichever was the most anterior. Treatment was given from Monday to Friday for 5 weeks.

**Figure 1:**
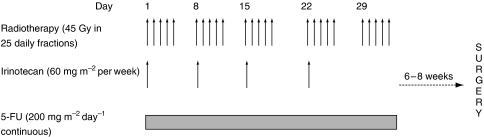
Patient treatment. 5-FU=5-fluorouracil.

5-FU 200 mg m^−2^ per day was administered by continuous iv infusion 7 days/week throughout radiotherapy. Irinotecan (50–70 mg m^−2^) was administered on days 1, 8, 15 and 22 via a 30-min iv infusion (day 1 being the first day of radiotherapy; Figure 1).

During phase I of the study, cohorts of three patients received irinotecan in a dose escalating between 50 and 70 mg m^−2^. Development of grade III and IV toxicity guided the calculation of the maximum-tolerated dose. Toxicity assessments were made according to the National Cancer Institute Common Toxicity Criteria version 3.0. In the event of non-haematological toxicity, the dose was modified as follows: for grade I toxicity, no treatment modification was made; for grade II toxicity, 5-FU and irinotecan were interrupted until resolution to grade 0, then resumed at full dose; for grade III toxicity, 5-FU and irinotecan were interrupted until resolution to grade 0, then resumed at 75% of the initial dose. For patients with grade II or grade III toxicity, radiotherapy was continued, but reviewed daily. For those with grade IV toxicity, 5-FU and irinotecan were discontinued and radiotherapy was interrupted; if symptoms did not resolve to grades 0–I within 2 weeks, radiotherapy was permanently discontinued.

Total mesorectal excisional (TME) surgery was planned 6–8 weeks after the completion of CRT in all patients. Note that only 28 of 31 had surgery, however, since two developed metastatic disease and one had a myocardial infarction prior to surgery.

### Tumour response

Every patient's treatment response was measured clinically and pathologically. Tumours were staged clinically, according to the TNM (tumour, node, metastasis) system from both pre-surgical and post-surgical CT/MRI scans. Tumour downstaging was considered complete when all lesions disappeared, and partial when tumour size decreased by more than 30%. Disease was considered to be progressive if tumour size increased by more than 20% (maximum transaxial or craniocaudal dimension). Stable disease was defined as tumours that decreased by less than 30% or increased by less than 20%, using the RECIST criteria (response evaluation criteria in solid tumours) (Therasse *et al*, 2000).

Patients were seen at least once a week during CRT, then weekly for 6 weeks after the end of radiotherapy.

Tumours that were resected after CRT were also classified by histopathology. The presence of residual disease macroscopically at surgery and microscopically in terms of tumour involvement (or clearance) at the CRM was used to classify the surgical resection as R_2_ (macroscopic residual disease), R_1_ (microscopic residual disease—CRM involved or disease within 1 mm of the CRM) or R_0_ (confirmed clear margins—CRM negative). A pathological complete response was recorded when no tumour was seen. Tumours in which only a few abnormal cells were seen without a residual tumour mass were considered indicative of microfocal residual disease. Pathological Dukes’ staging was also recorded.

After surgery, patients were followed up by the treating surgeon, according to the local protocol, at a maximum interval of every 6 months; serial carcinoembryonic antigen estimation was performed for up to 5 years. CT scanning of the abdomen and pelvis was also carried out at 6, 12 and 24 months following surgery.

### Statistical analysis

The probabilities of disease-free and overall survival were calculated using the Kaplan−Meier method. Disease-free and overall survival rates were calculated from the date of registration to the time of death or relapse. Disease-free survival was defined as the absence of relapse or recurrence of the primary cancer or death from any cause.

## RESULTS

### Dose escalation and patient characteristics

Twelve patients were recruited for phase I of the study. In the first cohort of three patients, who received irinotecan 50 mg m^−2^, there was one episode of grade III diarrhoea. Three more patients were enrolled for treatment with this dose, and none experienced a grade III or grade IV dose-limiting toxicity (DLT). There was also no DLT in the cohort of three patients who received irinotecan 60 mg m^−2^. However, two of the three patients in the cohort receiving irinotecan 70 mg m^−2^ experienced a DLT.

For phase II of the study, data were collected for 31 patients (23 (74%) men, eight (26%) women) aged 37–76 years (mean 61.8 years), with inoperable rectal cancer. All had threatened CRM and clinically fixed tumour as judged by examination under anaesthetic and CT and/or MR imaging. In all 20 patients had tumours 0–5 cm from the anal verge, six 5–10 cm and five 10–15 cm. They were treated with the maximum tolerated dose of irinotecan, which was judged to be 60 mg m^−2^ on the basis of results from the phase I trial.

### CRT administration and tolerability

One patient requested discontinuation of chemotherapy after 4 weeks of treatment, having previously required reductions in the irinotecan dose, but completed radiotherapy and subsequently underwent surgery. The other thirty (97%) patients received the prescribed four doses of irinotecan. Patients who completed the treatment protocol received a mean of 34.3 days (range, 28–35 days) of continuous iv 5-FU infusion. The 5-FU dose was reduced in three (10%) patients; in two of these patients, the dose was reduced because of adverse events (pyrexia and diarrhoea) and in one the dose reduction was the result of an administration error.

A mean radiation dose of 44.7 Gy (range, 37.8–45.0 Gy) was administered to patients who completed treatment. Only two patients had their total radiotherapy dose reduced. The toxicity observed was generally mild. The most frequent grade III toxicities experienced by the cohort were diarrhoea (*n*=4 (13%)) and constipation (*n*=2 (6%)). Only one (3%) patient experienced grade IV toxicity, which was fever without infection. The incidence of grade III and grade IV toxicities experienced by patients during treatment is given in Table 1. One patient died from an unrelated myocardial infarction approximately 4 weeks post-treatment and two patients were diagnosed with metastatic disease shortly after completing the full treatment protocol.

Twenty-eight patients underwent surgery. One patient died in the immediate post-operative period from an anastomotic leak; 27 patients were evaluable for follow-up after resection.

### CRT efficacy

All of the patients recruited underwent staging before treatment (*n*=31). In 21 (68%) patients the tumour was classified as T3, and in 10 (32%) as T4. Eleven (35%) patients had no nodal metastases (N0), 15 (48%) were staged as N1 and five (16%) were staged as N2.

Before surgery, 24 of 28 (86%) patients underwent repeat staging with pelvic MRI. Of these, seven (29%) were judged to have a complete clinical response, 12 (50%) a partial clinical response and four (17%) stable disease. One (4%) patient showed progressive disease on this MRI scan. Therefore, 19 of 24 (79%) patients assessed by MRI before surgery had downsized tumours on clinical staging.

In total, 28 of 31 (90%) patients underwent surgical resection and 27 of 31 (87%) had complete macroscopic resections. Surgery was performed 6–8 weeks after the end of radiotherapy. From the macroscopic and microscopic histopathology reports, patients’ residual disease was summarised as R_0_ in 22 of 27 (81%) patients, R_1_ in four (15%) patients and as R_2_ in one (4%) patient.

Histological staging of the evaluable patients after surgery revealed five of 27 (19%) patients in whom there was a pathological complete response. Four of the 27 (15%) patients had minimal residual (microfocal) disease. Eight of the 27 (30%) patients had Dukes’ stage B disease and 10 (37%) patients had Dukes’ stage C disease.

**Figure 2:**
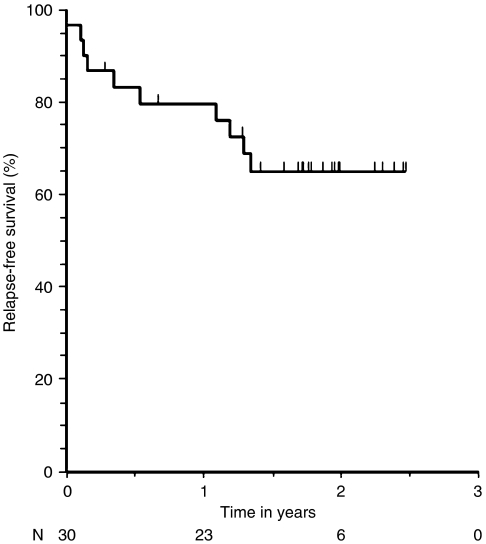
Relapse-free survival in patients who underwent surgical resection. The curve begins below 100% survival at time 0 because one patient died in the immediate post-operative period and was considered to have died without ever being disease-free.

**Figure 3:**
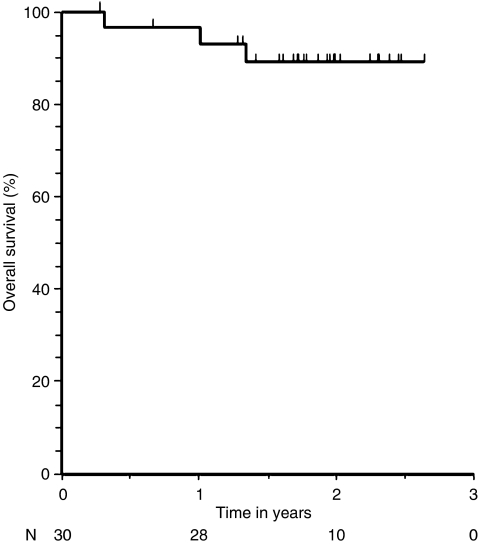
Overall survival in patients who underwent surgical resection.

After a median follow-up of 24 months (range, 8–43 months), 11 of 31 (35%) patients who were enrolled had experienced disease progression (two of these occurred pre-operatively) and three (10%) patients had died of their disease. There were five local recurrences in the pelvis—two with distant spread beyond the pelvis and three with local recurrence. Six (19%) patients relapsed with metastatic disease only. Of the patients who died, one death was the result of local progression of rectal cancer without distant spread, and two deaths were due to metastatic disease without local relapse. The estimate for 3-year relapse-free survival was 65% (Figure 2), and the estimate for 3-year overall survival was 90% (Figure 3).

## DISCUSSION

The results of this study suggest that even the most locally advanced rectal cancer can be downstaged and rendered operable, or even cured, by use of an appropriate CRT regimen. Furthermore, the short-term and long-term morbidity of irinotecan in combination with 5-FU and radiotherapy is acceptable, and follow-up has revealed excellent local and distant disease control. Other studies have not produced such good results with such low toxicity; however, this is a phase I/II study with carefully selected patients, and further work is needed to confirm these preliminary findings.

Of 31 patients with non-resectable stage T3/T4 rectal cancer, 28 (90%) underwent TME surgical resection of their tumour following treatment with irinotecan, 5-FU and radiotherapy. Complete surgical resection (R_0_ or R_1_) was achieved in 26 patients (84% of the initial 31, or 96% of all those who were assessable post-surgery). The R_0_ rate for all patients was 22/31 (71%), or 22/27 (81%) of those assessable post-surgery.

Phase II trials rarely include a comparator group, making the results from a single study difficult to assess in isolation. While comparisons between studies are limited because of inherent variability in patients, disease severity and treatments, an assessment of the relative efficacy and tolerability of a treatment is nevertheless helpful for determining whether a regimen offers potential benefits, justifying further study.

Prior to this study, it was thought that the problematic side effect of diarrhoea associated with each of irinotecan, 5-FU and radiation would cause severe toxicity should the agents be combined, rendering the regimen too toxic for routine use. However, the rate of grade III/IV toxicity (10%) noted in this study is comparable with that reported for oxaliplatin regimes (18–30%) (Alonso *et al*, 2004, 2007; Casado *et al*, 2004; Tucci *et al*, 2004; Fakih *et al*, 2005; Machiels *et al*, 2005; Avallone *et al*, 2006; Rutten *et al*, 2006; Rödel *et al*, 2007). Indeed, the findings of this study suggest a safety profile for irinotecan, 5-FU and radiotherapy that is comparable with previously reported findings with capecitabine and radiotherapy (rate of grade III/IV diarrhoea 12–28%) (Mehta *et al*, 2003; Navarro *et al*, 2003; Mitchell *et al*, 2004; Mohiuddin *et al*, 2006; Willeke *et al*, 2007), but using a lower radiation dose (45 Gy as opposed to 50.4 Gy in the capecitabine regimen).

Furthermore, the resection rate achieved in this study is higher than that observed in studies using radiotherapy alone (range, 64–75%) (Emami *et al*, 1982; Frykholm *et al*, 2001) or in some studies of CRT regimens (62–95%) (de la Torre *et al*, 1999; Frykholm *et al*, 2001; Klautke *et al*, 2006; Glynne-Jones *et al*, 2007), and it is comparable to that cited in other reports of research with irinotecan (90–100%) (Minsky *et al*, 1999; Mitchell *et al*, 2004; Klautke *et al*, 2005; Sebag-Montefiore *et al*, 2005). It is worth noting, that this current trial included only patients with T3/T4 cancers—that is patients with more advanced disease than those included in previously published studies.

Clinical staging in this study was performed by CT/MRI in a subset of patients after CRT treatment. At the time that the study was conducted, CT/MRI was used for investigational purposes only and was, therefore, not undertaken in all of the patients. Clinical staging revealed a complete clinical response rate of 29, and 79% of patients were judged to have downstaged tumours on MRI after treatment. Few studies have reported MR downstaging following CRT, although in a previous study of 31 patients treated with 5-FU and radiotherapy the tumour volume was decreased by at least half in 77% of patients (Glynne-Jones *et al*, 2007).

The proportion of patients obtaining a pathological complete response (19%) was similar to that reported for some other irinotecan-based protocols (11–25%) (Mitchell *et al*, 2004; Klautke *et al*, 2005; Sebag-Montefiore *et al*, 2005). Considering all irinotecan-based protocols together, the pathological complete response seems to be greater than that observed with radiotherapy alone (4%) (Frykholm *et al*, 2001) and comparable with most other fluoropyrimidine CRT regimens (12–28%) (de la Torre *et al*, 1999; Freyer *et al*, 2001; Frykholm *et al*, 2001; Carraro *et al*, 2002; Gérard *et al*, 2003; Aschele *et al*, 2005; Loi *et al*, 2005; Avallone *et al*, 2006; Klautke *et al*, 2006; Pucciarelli *et al*, 2006; Ryan *et al*, 2006). Four (15%) patients in the current study had microfocal residual disease, meaning overall that nine (29% of the phase II patient population) patients had a complete, or almost complete, response to treatment.

Considering CRM involvement, 81% of patients in this study who underwent TME surgery were classed as R_0_ after surgery (CRM-negative). In studies that report data on the number of CRM-negative cases, results range from 61–74% with radiotherapy (Emami *et al*, 1982; Frykholm *et al*, 2001) to 72–89% with 5-FU-based protocols (de la Torre *et al*, 1999; Frykholm *et al*, 2001; Avallone *et al*, 2006; Klautke *et al*, 2006; Glynne-Jones *et al*, 2007) or 88–89% when irinotecan is given as part of the CRT regimen (Klautke *et al*, 2005; Sebag-Montefiore *et al*, 2005). The addition of irinotecan to CRT regimens does not, therefore, seem to increase the high rate of CRM-negative surgery reported previously, but CRT certainly seems to be more successful than radiotherapy alone.

It should be noted that many of the studies mentioned above did not use CT/MRI to assess CRM involvement pre-treatment, but relied on clinical examination alone. Patients with early-stage tumours may, therefore, have been classified as CRM-negative, resulting in overestimation of this parameter.

A negative CRM may provide an early surrogate test of efficacy and a predictor of local recurrence, enabling the rapid evaluation of the many new treatment regimens currently being investigated (Cunningham *et al*, 2002). Patients who respond to pre-operative chemo-radiation have improved disease-free and overall survival compared with non-responders (Janjan *et al*, 2001). Therefore, the histological complete response rate and the impact of neoadjuvant therapy on clinical staging are other potential short-term end points that may be early indicators of disease-free and overall survival.

Comparison of the disease-free and overall survival results observed here with those of previous studies is complicated by the disease severity at recruitment, the duration of follow-up and whether second colorectal cancers or deaths due to causes other than rectal cancer are included. The only previous study of patients with rectal cancer receiving CRT with irinotecan and 5-FU reporting survival results was in patients with locally advanced rectal cancer who received neoadjuvant therapy. The 4-year disease-free and overall survival rates were 73 and 66%, respectively (Klautke *et al*, 2005). CRT in patients with non-resectable rectal cancer has been shown to significantly improve disease-free survival compared with radiotherapy alone, and may increase overall survival (Frykholm *et al*, 2001). Overall survival rates following CRT treatment with 5-FU have been reported to range from 26 to 73% (de la Torre *et al*, 1999; Frykholm *et al*, 2001; Avallone *et al*, 2006; Klautke *et al*, 2006; Glynne-Jones *et al*, 2007). Reported rates of disease-free or relapse-free survival range from 54 to 74% (de la Torre *et al*, 1999; Frykholm *et al*, 2001; Avallone *et al*, 2006; Klautke *et al*, 2006; Glynne-Jones *et al*, 2007), but again the studies vary in duration of follow-up. The overall and disease-free survival rates observed in the current study are, on the whole, greater than those seen previously. A prospective, randomised trial is now needed to confirm in larger numbers the impact on survival of adding irinotecan to CRT with 5-FU in patients with non-resectable rectal cancer.

Further work also continues to investigate whether using oral fluoropyrimidine capecitabine in place of 5-FU in the regimen can produce acceptable toxicity rates, especially with respect to diarrhoea (Gollins *et al*, 2006). Once complete, a prospective, randomised phase III study is proposed, comparing irinotecan 5-FU and radiotherapy with (i) irinotecan, capecitabine and radiotherapy and (ii) oxaliplatin, 5-FU and radiotherapy. This randomised trial will identify the most effective regimen and provide important information on whether R_0_ resection rates and pathological complete remission rates predict long-term survival.

When combined with 5-FU and radiotherapy, irinotecan appears to be both effective and safe for the neoadjuvant treatment of rectal cancer. The findings published here suggest that irinotecan has a place in CRT regimens for locally advanced rectal cancer. The regimen should now be compared with other non-irinotecan-based CRT regimens in a randomised phase III trial.

## Figures and Tables

**Table 1:** Grade III and Grade IV toxicities experienced by patients during treatment

**Toxicity**	**Grade III, *n* (%)**	**Grade IV, *n* (%)**
Diarrhoea	4 (13)	0 (0)
Constipation	2 (6)	0 (0)
Skin sores	1 (3)	0 (0)
Lethargy	1 (3)	0 (0)
Infection	1 (3)	0 (0)
Abdominal cramping	1 (3)	0 (0)
Chest pain	1 (3)	0 (0)
Fever (without infection)	0 (0)	1 (3)
Grade III/IV neutropenia	0 (0)	0 (0)
